# Learning in a Virtual Environment to Improve Type 2 Diabetes Outcomes: Randomized Controlled Trial

**DOI:** 10.2196/40359

**Published:** 2023-04-20

**Authors:** Constance M Johnson, Gail D'Eramo Melkus, Louise Reagan, Wei Pan, Sathya Amarasekara, Katherine Pereira, Nancy Hassell, Sarah Nowlin, Allison Vorderstrasse

**Affiliations:** 1 Cizik School of Nursing University of Texas Health Science Center at Houston Houston, TX United States; 2 School of Nursing Duke University Durham, NC United States; 3 Rory Myers College of Nursing New York University New York, NY United States; 4 School of Nursing University of Connecticut Storrs, CT United States; 5 Department of Nursing Mount Sinai Hospital New York, NY United States; 6 Elaine Marieb College of Nursing University of Massachusetts Amherst Amherst, MA United States

**Keywords:** eHealth, randomized controlled trial, self-management, type 2 diabetes mellitus, computer-mediated environments, virtual environment

## Abstract

**Background:**

Given the importance of self-management in type 2 diabetes mellitus (T2DM), a major aspect of health is providing diabetes self-management education and support. Known barriers include access, availability, and the lack of follow through on referral to education programs. Virtual education and support have increased in use over the last few years.

**Objective:**

The purpose of the Diabetes Learning in a Virtual Environment (LIVE) study was to compare the effects of the LIVE intervention (educational 3D world) to a diabetes self-management education and support control website on diet and physical activity behaviors and behavioral and metabolic outcomes in adults with T2DM over 12 months.

**Methods:**

The LIVE study was a 52-week multisite randomized controlled trial with longitudinal repeated measures. Participants were randomized to LIVE (n=102) or a control website (n=109). Both contained the same educational materials, but the virtual environment was synchronous and interactive, whereas the control was a flat website. Data were collected at baseline and 3, 6, and 12 months using surveys and clinical, laboratory, and Fitbit measures. Descriptive statistics included baseline characteristics and demographics. The effects of the intervention were initially examined by comparing the means and SDs of the outcomes across the 4 time points between study arms, followed by multilevel modeling on trajectories of the outcomes over the 12 months.

**Results:**

This trial included 211 participants who consented. The mean age was 58.85 (SD 10.1) years, and a majority were White (127/211, 60.2%), non-Hispanic (198/211, 93.8%), married (107/190, 56.3%), and female (125/211, 59.2%). Mean hemoglobin A_1c_ (HbA_1c_) level at baseline was 7.64% (SD 1.79%) and mean BMI was 33.51 (SD 7.25). We examined weight loss status versus randomized group, where data with no weight change were eliminated, and the LIVE group experienced significantly more weight loss than the control group (*P*=.04). There were no significant differences between groups in changes in physical activity and dietary outcomes (all *P*>.05), but each group showed an increase in physical activity. Both groups experienced a decrease in mean HbA_1c_ level, systolic and diastolic blood pressure, cholesterol, and triglycerides over the course of 12 months of study participation, including those participants whose baseline HbA_1c_ level was 8.6% or higher.

**Conclusions:**

This study confirmed that there were minor positive changes on glycemic targets in both groups over the 12-month study period; however, the majority of the participants began with optimal HbA_1c_ levels. We did find clinically relevant metabolic changes in those who began with an HbA_1c_ level >8.6% in both groups. This study provided a variety of resources to our participants in both study groups, and we conclude that a toolkit with a variety of services would be helpful to improving self-care in the future for persons with T2DM.

**Trial Registration:**

ClinicalTrials.gov NCT02040038; https://clinicaltrials.gov/ct2/show/NCT02040038

## Introduction

### Background

Diabetes affects 34 million US adults, most of whom have type 2 diabetes mellitus (T2DM) [1]. Achieving recommended glycemic targets in T2DM have been found to effectively reduce diabetes-related morbidity and mortality [2-4]. However, diabetes remains the seventh leading cause of death in the United States, and diabetes-related complications are the leading cause [5]. Self-management is integral to the control of T2DM since persons with diabetes provide the majority of their own care [6]. Evidence-based strategies for effective self-management include diabetes self-management education and support (DSME/S); an emphasis on the person’s role in managing diabetes; ongoing support from peers and professionals; strategies for coping with the demands of chronic illness; and collaborative care planning, goal setting, and problem-solving by a multidisciplinary team [6].

Diabetes self-management interventions have shown improvements in diabetes knowledge, self-management behaviors, and glycemic targets; however, the interventions and their effects have been relatively short-lived [7,8]. The most effective interventions have incorporated interactive, frequent, and somewhat individualized interactions between educators, providers, and persons with diabetes [9-11]. These interactive health interventions are thought to exert effects by enhancing knowledge, self-efficacy, problem-solving or coping skills, and social support, supporting persons with diabetes to manage their health behaviors and leading, in turn, to changes in clinical outcomes [12-14]. Growing evidence has revealed the need to improve ongoing self-management after DSME/S, including assistance with psychosocial issues that individuals with diabetes face, the improvement of peer-to-peer interactions and family or community support, and frequent educational content refreshers [6,11,15]. However, frequent and long-term interactions are costly, present difficulties for participant scheduling and attrition, and are not sustainable.

Further, in the current health care system, many persons with T2DM face barriers to receiving even minimal self-management support such as short-term DSME/S. Participation in face-to-face diabetes classes and group DSME/S sessions is low [16]; of people referred to DSME/S, only 66% report attending, whereas many are not even referred [17]. The Institute of Medicine has called for a redesign of health care systems to address this need for continuous patient-provider relationships in chronic disease [18]. Ongoing DSME/S are needed in a format that is sustainable in health care systems [6,19]. Widespread and low-cost internet access is bringing the potential of advanced web technologies to all, despite geography, economic status, and demographic factors [20,21]. According to 2019 census data estimates, 93% of American households own one or more computing devices [22], and 93% of US adults currently use the internet [23]. eHealth programs link people to support from peers and friends, particularly others with the same chronic disease or concerns, and to providers who can facilitate access to evidence-based information in a timely manner [24-26]. A Cochrane Review of eHealth programs concluded that users of these programs perceive higher levels of social support, are more knowledgeable, and have better behavioral and clinical outcomes than nonusers [13]. Person-centered information technology holds the promise of supporting the self-management of chronic diseases and potentially increasing the use of health care [27].

Internet interventions used to address T2DM self-management have resulted in increased support [15,28,29], self-efficacy [30-32], patient activation [33], and knowledge [14,31,34-36]; decreased health-related distress [32,33,37,38] and depressive symptoms [39]; improvements in glycemic targets [40,41] and self-management behaviors [30,33,42]; and more efficient use of primary care services [28], with decreased hospitalizations and emergency department visits [43,44]. The most effective DSME/S internet programs provide relevant content, engaging interactive elements, tailored personalized learning experiences, and self-assessment tools for monitoring and feedback [11,45]. The effects of internet and technological interventions on glycemic targets and self-management, however, vary from small to large and are frequently short term, due to a lack of interactivity in these interventions [46] and a drop-off in the use of some technologies over time by both clinicians and patients [15,42,47-50]. The drop-off may be related to the limited interactivity and static information [51].

A virtual environment (VE) enables accessing both synchronous and asynchronous diabetes education, skill-building activities, and peer or professional support from a home computer, opening doors to self-management education for many who face barriers (eg, location and time) to attending traditional clinic-based programs. VEs are computer-generated 3D representations of a simulated community on the internet. The users of this environment experience realism through feeling similar to being really there, which is also called presence [52]. Users self-represent as avatars and interact with other avatars or bots (computer-simulated robots mimicking human behavior) through voice or text chat and navigate from one location to another. Avatars can simulate human-like movement and gestures. These information spaces have the potential to be useful in providing not only information but also opportunities for users to practice new behaviors in real-life scenarios. A VE can also place DSME/S in a format that is easier to scale up once it is developed and tested in studies such as the proposed randomized controlled trial (RCT), ensuring sustainability and access [53]. Although the efficacy of a VE in providing ongoing education and support in chronic disease self-management has not been tested to date, we developed and established the feasibility of a virtual diabetes community that provides ongoing peer and professional support for persons with T2DM [54].

The social cognitive theory, characteristics of VEs, and the social-ecological model of social support [55] were used to guide this virtual intervention and have been described elsewhere [56] for our previous VE intervention. The social cognitive theory emphasizes the importance of cues to action, self-efficacy, and skill development in producing health behavior change [57-59]. The Learning in a Virtual Environment (LIVE) intervention allowed for the modeling of behaviors by health professionals and allowed participants to learn from each other in terms of behaviors modeled in their peer-to-peer interactions. Facilitated self-directed goal setting aided participants in identifying realistic behavior change goals. Participants in this intervention identified goals with health professionals and received ongoing feedback related to the achievement of those goals through synchronous classes, gamification, and Fitbit data (Fitbit, Inc).

### Objective

The purpose of the Diabetes LIVE study was to compare the effects of the LIVE intervention on diet (fat, fiber, fruit, and vegetable intake) and physical activity behaviors (minutes of moderate physical activity per week), behavioral outcomes (foot care, blood glucose monitoring, and medication adherence), as well as metabolic outcomes (glycated hemoglobin [HbA_1c_] level, lipids, blood pressure [BP], BMI, and waist circumference) in adults with T2DM over 12 months compared to a DSME/S control website (WebControl). We hypothesized that the active interaction in LIVE group for synchronous education and support would be the component that leads to effective change between the groups, as compared to the WebControl group who received asynchronous and flat modes of engagement (never in person). We hypothesized that dietary and physical activity behaviors, behavioral outcomes, and metabolic outcomes would improve over 12 months, with a higher rate of improvement in the LIVE intervention group. We also conducted a subgroup analysis (HbA_1c_ level >8.6%) to determine who would benefit from this type of intervention.

## Methods

### Study Design

The Diabetes LIVE study was a 52-week multisite (Duke University and New York University) parallel RCT with longitudinal repeated measures and an allocation ratio of 1:1. We evaluated the efficacy of the LIVE intervention compared to a DSME/S control website over 12 months. A full overview of the protocol was previously published [60]. The Diabetes LIVE protocol is registered at ClinicalTrials.gov (NCT02040038).

### Ethics Approval

Approval from both the Duke University (Pro00043325) and New York University (i14-00897) institutional review boards were obtained prior to participant recruitment.

### Randomization

Enrolled participants who provided informed consent were randomly assigned to 12 months of access to either the LIVE site or our WebControl site. Allocation to the intervention groups was made equally to each group at each site using block randomization. The synchronous LIVE site was a VE for DMSE/S with Fitbit physical activity monitoring, synchronous diabetes classes led by diabetes educators, and access to a forum for asynchronous discussion of diabetes-related topics with other participants (in the LIVE group) and diabetes educators [53,60,61]. The asynchronous WebControl site provided prerecorded American Diabetes Association self-management education classes with Fitbit physical activity monitoring, access to informational resources similar to the LIVE site, access to a forum for asynchronous discussion of diabetes-related topics with other participants in the WebControl group and diabetes educators, and email access to the diabetes educators. The email access to a diabetes educator for the WebControl group was intended to enhance usual care for 2 purposes: to reduce attrition by promoting improved follow-up assessment completion and to provide equal attention given that the intervention group could interact with experts in the VE.

### Participants

Eligible participants included those who were diagnosed with T2DM, were at least 21 years old, were able to read and write in English, had access to a personal computer with broadband internet connection in a private location (home), and were accessible by telephone. Participants could not have any preexisting medical condition(s) or severe diabetes-related complications that would interfere with study participation (eg, renal failure, stage III hypertension [BP >180/110], severe orthopedic conditions or joint replacement scheduled within 6 months, paralysis, bleeding disorders, or cancer). They could not currently be taking any anticoagulant medications such as warfarin. They had to be able to travel to a clinical laboratory for blood work. Participation was not restricted by baseline HbA_1c_ level or prior diabetes self-management education participation. The evidence and recommendations show that persons living with diabetes need DSME/S at multiple time points in their diabetes trajectory, including at least annually and when key transitions or challenges in the treatment plan occur, and that DSME/S are essential to maintaining glycemic targets [16].

### Recruitment

The recruitment strategies and recruitment outcomes for this RCT have been described elsewhere [62]. Overall, we recruited the participants from a variety of sources, including clinics, study registries, community and print advertisements, research networks, media such as radio, social networking sites, hospital databases, and participants who consented in prior studies. Participants were recruited from North Carolina and southern New England including New York.

### Intervention

The LIVE intervention was developed using an iterative design approach that was informed by the researchers, study team, the programmers, and participants with T2DM [53]. The VE was designed as a virtual community to provide DSME/S and was based on our previous successful Second Life Impacts Diabetes Education & Support (SLIDES) pilot intervention [54,63]. A full description of the LIVE intervention has been previously published [53]. In summary, nurse practitioners, diabetes educators, and nutritionists held twice weekly classes using the American Diabetes Association and American Association of Diabetes Educators self-management education curriculum [64]. The educator team provided both the synchronous classes in the LIVE site and the asynchronous, recorded content in the WebControl site with no differentiation in the team members for the control and intervention sites. In addition to this standard content, the diabetes educators developed 12 more classes focused on more advanced content [61]. Participants self-represented as avatars with anonymous names. However, the research coordinators kept track of the real names and associated anonymous names in a password-protected database. All of the diabetes educators knew the names of each participant by their anonymous name and thus would have been aware of multiple identities. Additionally, each anonymous name was associated in the username-password database in the backend, and this prevented unidentified participants from entering the environment. The environment appeared to be a town with numerous informational resources embedded within businesses (ie, pharmacy, bookstore, restaurant, grocery store, etc). In addition, a number of games and principles of gamification were used to assist with participant interaction with the environment [53]. Interaction within the LIVE site was synchronous with all participants using voice communication (by headset with a microphone).

The WebControl site was an asynchronous password-protected website with the same educational content as the VE, but participants had no direct interaction via voice with the other participants, the diabetes educators, nurse practitioners, or nutritionists [60]. Only the study participants and study team had access to the site. The same diabetes education classes in the LIVE site were posted at the same frequency as prerecorded modules by the educators. The difference between the control and intervention groups were the presentation of information (one in a 3D VE and the other in a flat website) and the immersion and realism (ability to interact with 3D objects and avatars) in the LIVE intervention. See Table 1 for an outline of the components on each site.

**Table 1:** Comparison of components in the virtual environment (VE) versus the control website (WebControl) group.

Component	LIVE^a^	WebControl
Platform	Password-protected access to the secure VE built with Epic Games Unreal Engine 3.	Password-protected access to a secure website, only accessible to participants randomized to the website within the study.
Classes based on American Diabetes Association curricular standards	Biweekly classes held synchronously for 26 weeks were taught by diabetes educators. All classes were taped and participants could go back and listen to prior classes. Classrooms were designed to resemble a classroom with chairs, a stage, and a screen to post slides. Diabetes educator presented the content as an avatar.	The same lectures in the LIVE site were recorded and posted by diabetes educators. Participants could listen at any time to the posted lectures.
Self-representation	An avatar with an anonymous name.	Website access with an anonymous name.
Fitbits	Provided and tracked physical activity.	Provided and tracked physical activity.
Forum	Participants were able to post, ask questions, and discuss weekly content with other participants randomized to the VE and diabetes educators. It was an asynchronous message board.	Participants were able to post, ask questions, and discuss weekly content with other participants randomized to the WebControl group and diabetes educators. It was an asynchronous message board.
Game quests	Present and changed weekly in the site.	None.
Gamification	We posted the most interactive participants and most active Fitbit users on leaderboards monthly.	We posted the most interactive participants and most active Fitbit users on leaderboards monthly.
Voice	Participants were able to speak with diabetes educators and other participants via a headset at any time; both were in the site simultaneously.	Participants were unable to speak using voice with other participants or diabetes educators. Participants did not know when other participants were on the website, other than the forum.
Message and signs in the sites	Health promotion messages were posted in each location in the site and changed monthly.	Health promotion messages were posted on the home page and changed monthly.
Educational content	The educational content was posted in the gym (exercise and stress-relief videos), restaurants (chain and regional menus with expert feedback on items), the bookstore (links to buy books on the web and internet resources), the pharmacy (links to buy pharmacy items on the web), the clothing store (to buy clothing for the avatar with points earned through game playing), and grocery and convenience stores (the nutritional content of grocery store items and feedback were created by diabetes educators).	The educational content was posted in the gym (exercise and stress-relief videos), restaurants (chain and regional menus with expert feedback on items), the bookstore (links to buy books online, and internet resources), the pharmacy (links to buy pharmacy items on the web), and grocery and convenience stores (the nutritional content of grocery store items and feedback were created by diabetes educators).

^a^LIVE: Learning In a Virtual Environment.

### Study Procedures

The detailed study protocol was previously reported [60]. Briefly, once informed consent was obtained and the participants were randomly assigned to 1 of the 2 arms of the study, baseline data were obtained and the participants were taught how to use the LIVE site or the WebControl site. All participants were assigned an email account that was solely used to set up their Fitbit accounts, and they were given a headset with a microphone. Participants assigned to the LIVE site developed their avatar and their anonymous avatar name based on their preference. Written instructions for accessing either site were provided, and 3 days after their baseline appointment, participants received a follow-up telephone call to address questions about the study procedures and were provided a reorientation to their randomized site if necessary. Both sites were available to the participants through their username and password 24 hours/day, 7 days per week. Participants had access to their assigned site for 12 months.

Each participant was asked to sign into the site twice weekly for the first 3 months, followed by use at the participant’s discretion for the remaining 9 months. Participants were advised to consult their health care provider regarding any medication regimen changes or side effects, symptoms, or health status changes. Medical management was outside the scope of this intervention.

There were several potential risks to participants during study participation, including risks associated with venipuncture, physical activity, and data breaches. During the trial, the principal investigators met with the research staff weekly, the remote site principal investigator every 2-4 weeks as needed, and the data safety monitoring board annually. There were no adverse events associated with participation in this study or attributed to the interventions.

### Measures

#### Overview

Participants provided information on demographic characteristics (age, race/ethnicity, marital status, income level, education level, employment status, sex, the duration of diabetes, prior diabetes classes, and current medications). To meet our primary aims of assessing the effects of the LIVE intervention on physical activity, diet, behavioral and glycemic targets, and cardiovascular outcomes in adults with T2DM over 12 months, we measured these through REDCap (Research Electronic Data Capture; Vanderbilt University) surveys, in-person measurements, and blood samples as defined below.

#### Physical Activity and Diet

Physical activity was measured through the use of a Fitbit, which recorded the minutes of moderate physical activity per week. To date, studies using Fitbit have demonstrated good reliability and validity (especially if activity types are recorded) [65,66].

Dietary intake was assessed using the National Cancer Institute Multifactor Screener [67], a 16-item questionnaire to assess the frequency of intake of a variety of foods over the past month.

Self-report of behavioral outcomes was measured using the Summary of Diabetes Self-care Activities Scale. This scale measured foot care, blood glucose monitoring, exercise, and medication taking in terms of days per week. The instrument has good inter-item correlations within scales (mean *r*=0.47) and moderate test-retest correlations (mean *r*=0.40) [68].

#### Metabolic Measurements

Glycemic targets were measured as HbA_1c_ levels through central laboratory measures (Quest Labs and Labcorp) [69], indicating average glucose levels over the prior 3 months. BP, BMI (calculated from height and weight), and waist circumference were measured as clinical parameters associated with glycemic stability and diabetes-related complications (heart disease and stroke) that are influenced by self-management.

Lipid levels (low-density lipoprotein [LDL], high-density lipoprotein [HDL], total cholesterol, and triglycerides) were obtained from a clinical laboratory improvement amendments–certified central laboratory analysis (Quest Labs and Labcorp) of blood samples. These are indicators of cardiovascular disease risk, which is highly linked to diabetes [70].

#### Social Support

Social support was assessed using the Diabetes Support Scale, a 12-item survey specific to support in an internet-based diabetes intervention. This well-validated survey has an alpha of .90 to .93, sensitivity to intervention effects, and construct validity [29]. Diabetes support was measured at baseline and 3, 6, and 12 months.

### Data Collection

Survey data were collected and managed using REDCap [71], hosted on the university website. REDCap is a secure, web-based application designed to support data capture for research studies. Demographic data were captured by a self-report survey, and enrollment data were obtained from the screening and enrollment by the study coordinators at each site. Study coordinators collected weight and waist circumference during in-person visits. Fitbit data were downloaded from the participant’s Fitabase (Fitbit, Inc). Clinical data were measured by a predetermined local laboratory (for HbA_1c_ and lipid levels).

For this analysis, we are reporting on the baseline and 3-, 6-, and 12-month data from clinical and survey data. Data were cleaned by study coordinators, statisticians, and investigators. Data were verified with source documents by the study staff.

### Statistical Analysis

Our power analysis using the primary outcomes of differences between dietary intake and physical activity behaviors was 200 participants with an effect size of 0.50. This was our minimum sample target [62]. Descriptive statistics were first calculated to describe the sample characteristics, such as sociodemographics and diabetes history, as well as the primary outcomes by study arms. A few of the sample characteristics had missing data ranging from 10% (21/211) to 37.9% (80/211), and their descriptive statistics were nevertheless computed as is (see *Sample Characteristics* in the *Results* section) to show the true status of the sample data. All but one sample characteristic (ie, diabetes support) did not have significant differences between the intervention and control groups due to the randomization. Thus, only diabetes support was controlled as a covariate in all the subsequent statistical modeling.

The effects of the intervention on the outcomes were initially examined by comparing the means and SDs of the outcomes across the 4 time points (ie, 0, 3, 6, and 12 months) between study arms, followed by multilevel modeling (ie, mixed models) on the trajectories of the outcomes over 12 months. Multilevel modeling was appropriate for modeling longitudinal data with attrition, as we had in this study, because (1) multilevel modeling acts similar to an intent-to-treat analysis by including all participants with at least one data point [72,73] and (2) the maximum likelihood estimation in multilevel modeling automatically deals with missing data on time-varying outcomes during parameter estimation. Diabetes support was the only time-fixed covariate in the model. Although diabetes support had 10% (21/211) of missing data, the missing data were imputed by means before being included in multilevel modeling. All the analyses were conducted with SAS software (version 9.4; SAS Institute), and the significance level was set at .05.

## Results

### Sample Characteristics

**Figure 1:**
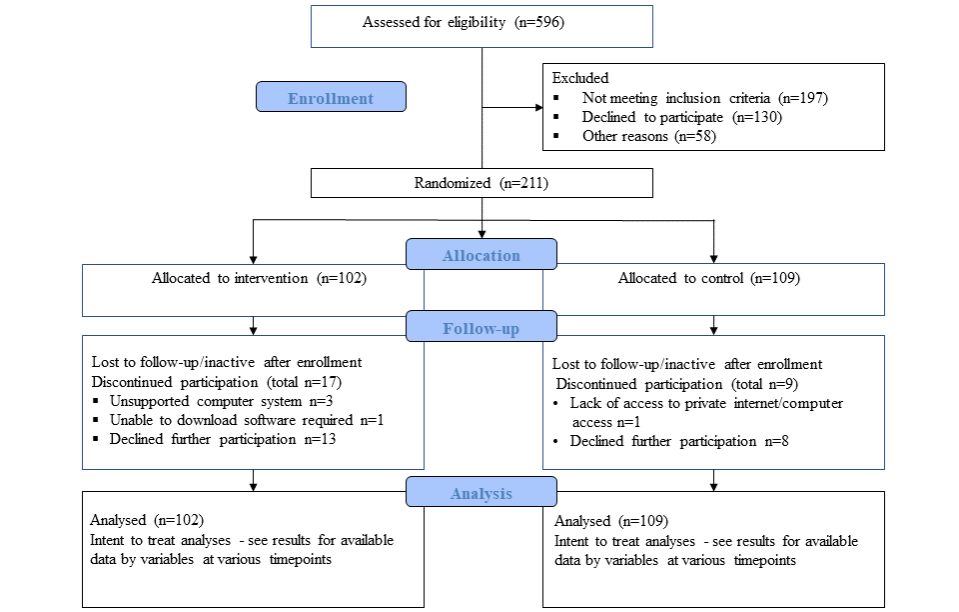
CONSORT diagram of diabetes LIVE (Learning in a Virtual Environment).

From August 2014 through September 2016, we had assessed for eligibility a total of 596 potential participants, 197 of whom were excluded as outlined in the CONSORT (Consolidated Standards of Reporting Trials) diagram (Figure 1). Of the 211 enrolled participants, 102 were randomized to the intervention group and 109 were randomized to the WebControl group at 2 sites—158 at Duke University and 53 at New York University [62].

Overall, the mean age of participants in the LIVE group was 58.85 (SD 10.1) years, and the majority were White (127/211, 60.2%), non-Hispanic (198/211, 93.8%), married (107/190, 56.3%), and female (125/211, 59.2%). A majority (108/189, 571.%) of participants had attained a bachelor’s degree, less than half (74/188, 39.4%) were employed full-time, and the majority (179/211, 84.8%) had moderate to high household income levels (see Table 2). The average duration of diabetes was over 10 years, with a median of 8 years. More than half (110/183, 60.1%) had participated in diabetes education classes in the past.

**Table 2:** Participant characteristics (n=211).

Characteristics		Total (n=211)	VE^a^ intervention group (n=102)	WebControl^b^ group (n=109)	*P* value^c^	
Age (years), mean (SD)		58.85 (10.14)	59.12 (10.62)	58.60 (9.72)	.71	
**Sex, n (%)**						.51
	Female	125 (59.2)	60 (58.8)	65 (59.6)		
	Male	86 (40.8)	42 (41.2)	44 (40.4)		
**Race, n (%)**						.21
	Asian	8 (3.8)	4 (3.9)	4 (3.7)		
	Black or African American	67 (31.8)	35 (34.3)	32 (29.4)		
	Other	9 (4.2)	5 (4.9)	4 (3.7)		
	White	127 (60.2)	58 (56.9)	69 (63.3)		
**Ethnicity, n (%)**						.33
	Hispanic	13 (6.2)	5 (4.9)	8 (7.3)		
	Non-Hispanic	198 (93.8)	97 (95.1)	101 (92.7)		
**Marital status^d^, n (%)**						.30
	Single	83 (43.7)	37 (41.1)	46 (46)		
	Married	107 (56.3)	53 (58.9)	54 (54)		
**Education^e^, n (%)**						.48
	High school or below	19 (10)	9 (10)	10 (10.1)		
	Some college or an associate degree	62 (32.9)	28 (31.1)	34 (34.3)		
	Bachelor’s degree or above	108 (57.1)	53 (58.8)	55 (55.5)		
**Employment^f^, n (%)**						.51
	Full-time	74 (39.4)	38 (42.2)	36 (36.7)		
	Part-time	28 (14.9)	10 (11.1)	18 (18.4)		
	Retired or not working	86 (45.7)	42 (46.7)	44 (44.9)		
**Household income, n (%)**						.49
	<24,999	32 (15.2)	12 (11.8)	20 (18.3)		
	25,000-69,999	98 (46.4)	49 (48)	49 (45)		
	>70,000	81 (38.4)	41 (40.2)	40 (36.7)		
Duration of diabetes (years)^g^, mean (SD)		10.84 (8.66)	11.05 (8.71)	10.61 (8.66)	.77	
Prior diabetes education^h^, n (%)		110 (60.1)	49 (55.1)	61 (64.9)	.11	
Diabetes support (range 12-84)^i^, mean (SD)		55.55 (18.30)	52. 47 (18.61)	58.32 (17.65)	.03	
**Diabetes medications (not mutually exclusive; all participants: n=211; intervention: n=102; control: n=109), n (%)**						
	Oral meds	148 (70.1)	73 (71.6)	75 (68.8)		.39
	Insulin	59 (28)	25 (24.5)	34 (31.2)		.18
	Other Meds	17 (8.1)	10 (9.8)	7 (6.4)		.26

^a^VE: virtual environment.

^b^WebControl: control website.

^c^*P* values from 2-tailed *t* tests for continuous variables and chi-square tests for categorical variables.

^d^Total: n=190; intervention: n=90; control: n=100.

^e^Total: n=189; intervention: n=90; control: n=99.

^f^Total: n=188; intervention: n=90; control: n=98.

^g^n=131.

^h^Total: n=183; intervention: n=89; control: n=94.

^i^n=190.

### Descriptive Statistics of Primary Outcomes Measures

The mean HbA_1c_ level at baseline was 7.64% (SD 1.79%) and the median was 7.10%, indicating that many had optimal HbA_1c_ levels at baseline with 28% (59/211) of the participants using insulin to manage their diabetes. Overall, both groups had good BP and lipid values at baseline, indicating that the recommended targets were met. Persons with high weight were prevalent with a mean BMI of 33.51 (SD 7.25) and a median BMI of 32.86 (range 18.07-58.67). Most participants (122/181, 67.4%) reported light to moderate physical activity levels, and dietary assessments revealed fat intake within the recommended range (mean 34.7%, SD 4.4%), with low fruit and vegetable intake (mean 4.3, SD 1.8 servings/day) and fairly low fiber intake (mean 16.7, SD 9.2 g). Table 3 outlines the primary outcome measures at baseline across both groups.

**Table 3:** Baseline primary outcome measures.

Variable	Value, mean (SD)	Value, median (range)
HbA_1c_^a^ (%)	7.64 (1.79)	7.10 (5.5-14.1)
Systolic blood pressure (mmHg)	136.96 (17.37)	136.50 (100-225)
Diastolic blood pressure (mmHg)	80.48 (9.94)	80 (53-108)
Total cholesterol (mg/dL)	167.01 (39.93)	162.50 (86-375)
LDL^b^ (mg/dL)	89.4 (32.33)	87.50 (17-260)
HDL^c^ (mg/dL)	50.54 (16.63)	47 (14-113)
Triglycerides (mg/dL)	138.97 (94.79)	116 (33-557)
Weight (kg)	93.88 (20.27)	92.35 (45.1-164.1)
BMI	33.51 (7.25)	32.86 (18.07-58.67)
Waist circumference (cm)	108.27 (16.18)	108 (38-156)
Exercise (days/week)	2.65 (2.16)	2.5 (0-7)
Fat intake (%)	34.74 (4.37)	33.90 (16.94-52.14)
Fiber intake (g)	16.69 (9.27)	14.22 (7.31-66.89)
Fruit and vegetable intake (servings/day)	4.29 (1.75)	3.99 (0 to ≥4)

^a^HbA_1c_: glycated hemoglobin.

^b^LDL: low-density lipoprotein.

^c^HDL: high-density lipoprotein.

### Effects of Random Group Assignment on Diet and Physical Activity

The mean number of days participants engaged in physical activity is shown in Table 4. Although changes in physical activity did not significantly differ between the LIVE and WebControl groups (*P*=.37), both groups had a mean increase in exercise over the 12 months. The number of days participants exercised increased on average from mean 2.63 (SD 2.01) days to mean 3.4 (SD 1.99) days in the LIVE group and from mean 2.67 (SD 2.27) days to mean 3.38 (SD 2.19) days in the WebControl group. We found the estimated differences in the slopes of trajectories from WebControl to LIVE to be –0.01. Comparing the trajectories over 12 months and controlling for diabetes support, we determined that changes in exercise did not differ significantly between the groups (*P*=.93).

With regard to physical activity, we found the estimated differences in the slopes of trajectories from WebControl to LIVE to be –0.04. Comparing the trajectories over 12 months and controlling for diabetes support, we determined that changes in physical activity did not significantly differ between the groups (*P*=.37).

The average daily participant steps measured by Fitbit increased over the 12 months in both study groups, although there were no statistically significant group differences (*P*=.29). The mean number of daily steps increased from 5569.84 (SD 4113.39) at baseline to 6532.00 (SD 4445.40) at 12 months in the WebControl group. In the LIVE group, the mean number of steps increased from 6153.20 (SD 3651.58) at baseline to 6347.31 (SD 3302.27) at 12 months.

With regard to dietary intake, the percent of energy from fat remained stable over the 12 months in both study groups (estimated differences=–0.15; *P*=.51). There were no statistically significant differences in fruit and vegetable intake between groups (estimated difference=–0.03; *P*=.73), and with regard to fiber intake, there was a steady increase in both groups at 6 months that decreased at 12 months (estimated differences=0.37; *P*=.41).

**Table 4:** Effects on diet and physical activity by study group.

Outcome and group		Baseline			3 months			6 months			12 months			*P* value^a^	
		Participant, n	Value, mean (SD)	Participant, n		Value, mean (SD)	Participant, n		Value, mean (SD)	Participant, n		Value, mean (SD)			
**Exercise (days/week)**															.93
	LIVE^b^	89	2.63 (2.01)	70		3.32 (2.28)	64		3.48 (1.90)	56		3.40 (1.99)			
	WebControl^c^	99	2.67 (2.27)	76		3.55 (2.30)	75		3.15 (2.14)	64		3.38 (2.19)			
**Physical activity (self-report)—exercise intensity**															.37
	LIVE	87	2.41 (0.91)	70		2.61 (1.00)	64		2.66 (0.98)	52		2.44 (1.00)			
	WebControl	94	2.41 (0.99)	72		2.56(1.06)	73		2.70 (0.97)	62		2.61 (1.08)			
**Fitbit (average daily steps)**															.29
	LIVE	90	6153.20 (3651.58)	N/A^d^		N/A	57		5749.81 (4112.12)	40		6347.31 (4445.40)			
	WebControl	101	5569.84 (4113.39)	N/A		N/A	66		6244.94 (3639.18)	49		6532.00 (4113.39)			
**Fat intake (%)**															.51
	LIVE	86	35.36 (4.39)	62		34.07 (3.02)	59		35.56 (6.07)	53		35.02 (4.16)			
	WebControl	90	34.13 (4.26)	66		35 (7.05)	68		34.59 (3.80)	53		34.93 (3.55)			
**Fiber intake (g)**															.41
	LIVE	86	16.20 (7.55)	62		18.77 (14.35)	59		19.06 (11.26)	52		17.85 (8.53)			
	WebControl	88	17.16 (10.66)	63		18.30 (10.54)	69		18.58 (9.03)	53		16.68 (9.25)			
**Fruit and vegetable intake (servings/day)**															.73
	LIVE	86	4.17 (1.58)	66		4.79 (2.14)	60		4.55 (1.67)	53		4.52 (1.85)			
	WebControl	93	4.40 (1.90)	70		4.93 (3.06)	71		4.73 (1.92)	57		4.35 (2.04)			

^a^The *P* values were calculated by comparing the trajectories over 0-12 months while controlling for diabetes support.

^b^LIVE: Learning in a Virtual Environment.

^c^WebControl: control website.

^d^N/A: not available.

### Effects of Random Group Assignment on Clinical Outcomes

As shown in Table 5, both groups experienced a decrease in mean HbA_1c_ levels, systolic and diastolic BP, cholesterol, and triglycerides over the course of 12 months of study participation. There were no statistically significant differences in any of these variables for the trajectories over 12 months between groups while controlling for diabetes support. However, changes in triglycerides between the 2 groups approached but did not have significance (*P*=.06). Changes in weight (*P*=.77), BMI (*P*=.99), and waist circumference (*P*=.80) differed by study group, but these were not significantly different. The WebControl group showed a decrease at 6 months but an increase at 12 months, whereas the LIVE group showed a consistent downward trajectory over the 12 months.

We explored weight change status by randomized group, where data with no weight change were eliminated, and we found that 48 of the LIVE participants gained (21/103, 20.4%) or lost (27/103, 26.2%) weight during the study and 55 of the WebControl group participants gained (35/103, 34%) or lost (20/103, 19.4%) weight during the study. The differences between the groups were statistically significant (chi-square), with the LIVE group experiencing more weight loss than the WebControl group (*P*=.04).

We additionally examined changes over time in behavioral outcomes (see Table 6). Although there were no statistically significant changes in the behavioral outcomes of foot care (*P*=.13), blood glucose monitoring (*P*=.59), or medication taking (*P*=.22), we did note clinical improvements in foot care in the LIVE group from a mean of 2.98 (SD 2.36) days per week to a mean of 4.05 (SD 2.38) days per week.

When we explored changes in clinical markers for participants whose HbA_1c_ level was elevated (8.6% or higher) at baseline, there were clinical improvements in both groups from baseline to 12 months in HbA_1c_ levels, total cholesterol, LDL, triglycerides, and waist circumference (see Table 7). There were no statistically significant differences between the groups for change between time points (all *P*>.05). This subanalysis was exploratory.

**Table 5:** Effects on glycemic and cardiovascular measurements by random group assignment.

Outcome and group			Baseline			6 months			12 months			Estimated differences in the slopes of trajectories from WebControl^a^ to LIVE^b^			*P* value^c^	
		Participant, n		Value, mean (SD)	Participant, n		Value, mean (SD)	Participant, n		Value, mean (SD)						
**HbA_1c_^d^ (%)**													0.08			.16
	LIVE	88		7.50 (1.58)	60		7.53 (1.49)	46		7.41 (1.36)						
	WebControl	93		7.73 (1.92)	65		7.50 (1.58)	52		7.29 (1.27)						
**Systolic blood pressure (mmHg)**													–0.72			.28
	LIVE	101		137.53 (17.9)	72		133.28 (12.86)	54		132.39 (17.06)						
	WebControl	109		136.69 (16.60)	74		134.61 (16.70)	60		135.15 (18.23)						
**Diastolic blood pressure (mmHg)**													–0.05			.90
	LIVE	101		81.18 (9.54)	72		80.57 (10.49)	54		79.57 (10.92)						
	WebControl	109		80.13 (10.38)	74		79.59 (11.44)	60		79.02 (11.41)						
**Total cholesterol (mg/dL)**													0.19			.89
	LIVE	88		164.82 (36.38)	58		166.76 (35.33)	44		156.00 (30.44)						
	WebControl	90		170.50 (42.51)	62		169.73 (38.35)	52		164.56 (36.45)						
**LDL^e^ (mg/dL)**													–0.82			.53
	LIVE	87		88.30 (25.77)	56		91.09 (29.44)	43		84.33 (25.05)						
	WebControl	85		90.12 (36.92)	61		94.70 (35.94)	52		92.14 (33.16)						
**HDL^f^ (mg/dL)**													0.59			.12
	LIVE	88		49.16 (15.09)	58		48.19 (14.07)	44		48.23 (16.11)						
	WebControl	92		50.65 (16.50)	61		50.31 (13.24)	52		48.12 (13.97)						
**Triglycerides (mg/dL)**													6.68			.06
	LIVE	88		134.47 (80.10)	58		147.67 (98.82)	45		130.56 (74.45)						
	WebControl	91		151.51 (119.2)	61		129.89 (54.17)	52		121.44 (53.02)						
**Weight (kg)**													–0.06			.77
	LIVE	101		94.96 (20.26)	72		94.02 (17.52)	54		91.74 (18.86)						
	WebControl	109		95.34 (23.04)	76		92.37 (21.84)	61		93.49 (21.81)						
**Waist circumference (cm)**													–0.13			.80
	LIVE	101		108.67 (16.54)	72		108.76 (13.54)	54		106.50 (14.89)						
	WebControl	109		109.11 (16.76)	75		106.25 (16.52)	60		107.10 (20.52)						
**BMI**													–0.00			.99
	LIVE	101		33.92 (7.21)	72		33.40 (6.18)	54		32.68 (6.43)						
	WebControl	109		33.45 (7.59)	76		32.93 (7.68)	59		33.08 (7.93)						

^a^WebControl: control website.

^b^LIVE: Learning in a Virtual Environment.

^c^The *P* values were calculated by comparing the trajectories over 0-12 months while controlling for diabetes support.

^d^HbA_1c_: glycated hemoglobin.

^e^LDL: low-density lipoprotein.

^f^HDL: high-density lipoprotein.

**Table 6:** Effects on behavioral outcomes and diabetes support by randomized group assignment.

Outcome and group		Baseline		3 months		6 months			12 months			Estimated differences in the slopes of trajectories from WebControl^a^ to LIVE^b^			*P* value^c^	
		Participant, n	Value, mean (SD)	Participant, n	Value, mean (SD)	Participant, n	Value, mean (SD)	Participant, n		Value, mean (SD)						
**Foot care (days/week)**													0.16			.13
	LIVE	89	2.98 (2.36)	71	3.57 (2.37)	64	3.91 (2.37)	55		4.05 (2.38)						
	WebControl	98	3.54 (2.46)	75	4.28 (2.12)	75	4.11 (2.36)	63		3.77 (2.32)						
**Blood glucose monitoring (days/week)**													0.06			.59
	LIVE	88	3.66 (2.67)	70	3.94 (2.58)	63	4.00 (2.59)	55		4.30 (2.77)						
	WebControl	98	3.77 (2.76)	76	4.16 (2.61)	75	4.26 (2.66)	63		3.96 (2.75)						
**Medication taking (days/week)**													0.14			.22
	LIVE	74	5.50 (2.28)	65	5.72 (2.15)	58	5.76 (2.29)	46		6.13 (1.77)						
	WebControl	88	5.67 (2.15)	68	6.19 (1.61)	69	5.45 (2.52)	60		5.57 (2.32)						

^a^WebControl: control website.

^b^LIVE: Learning in a Virtual Environment.

^c^The *P* values were calculated by comparing the trajectories over 0-12 months while controlling for diabetes support.

**Table 7:** Effects on glycemic and cardiovascular measures by random group and glycated hemoglobin (HbA_1c_) level >8.6% at baseline.

Outcome and group		Baseline			6 months			12 months			*P* value	
		Participant, n	Value, mean (SD)	Participant, n		Value, mean (SD)	Participant, n		Value, mean (SD)			
**HbA_1c_ (%)**												.29
	LIVE^a^	18	10.08 (1.37)	12		9.22 (1.67)	8		9.27 (0.97)			
	WebControl^b^	19	10.93 (1.83)	11		9.76 (2.62)	8		8.64 (0.98)			
**Systolic blood pressure (mmHg)**												.15
	LIVE	18	141.61 (14.61)	11		132.58 (11.84)	8		133.64 (21.79)			
	WebControl	19	127.68 (11.30)	11		124.45 (18.26)	9		135.22 (21.99)			
**Diastolic blood pressure (mmHg)**												.18
	LIVE	18	81.11 (7.78)	12		77.92 (8.91)	11		82.64 (10.08)			
	WebControl	19	77.84 (8.49)	11		77.64 (11.63)	9		78.00 (12.86)			
**Total cholesterol (mg/dL)**												.86
	LIVE	18	175.00 (37.93)	11		178.82 (48.89)	8		149.00 (28.42)			
	WebControl	18	166.95 (50.97)	11		167.73 (38.36)	7		161.86 (36.40)			
**LDL^c^ (mg/dL)**												.56
	LIVE	18	93.83 (30.47)	11		109.27 (37.07)	8		85.25 (20.41)			
	WebControl	17	96.76 (44.67)	11		93.73 (34.36)	7		95.43 (34.67)			
**HDL^d^ (mg/dL)**												.19
	LIVE	18	48.94 (14.04)	11		41.64 (10.08)	8		43.25 (14.21)			
	WebControl	19	45.00 (8.50)	11		46.73 (11.21)	7		46.00 (9.85)			
**Triglycerides (mg/dL)**												.48
	LIVE	18	161.17 (89.72)	11		147.45 (75.44)	8		103.75 (23.35)			
	WebControl	19	165.11 (132.69)	11		136.64 (49.20)	7		101.43 (36.16)			
**Weight (kg)**												.09
	LIVE	18	95.88 (19.99)	12		96.92 (15.95)	11		89.56 (23.74)			
	WebControl	19	94.20 (19.71)	12		91.69 (18.66)	9		97.42 (16.09)			
**Waist circumference (cm)**												.21
	LIVE	18	111.78 (16.35)	12		114.23 (12.03)	11		105.64 (18.08)			
	WebControl	19	109.60 (16.26)	12		104.46 (13.24)	9		107.39 (11.11)			
**BMI**												.09
	LIVE	18	34.63 (6.44)	12		34.86 (5.12)	11		32.19 (7.55)			
	WebControl	19	33.54 (7.23)	12		33.40 (7.80)	9		35.01 (8.24)			

^a^LIVE: Learning in a Virtual Environment.

^b^WebControl: control website.

^c^LDL: low-density lipoprotein.

^d^HDL: high-density lipoprotein.

## Discussion

### Principal Findings

In this 12-month VE intervention study, we investigated the effects of a diabetes VE (LIVE) versus a WebControl group on diet (percent of energy from fat, daily grams of fiber, and daily servings of fruits and vegetables), physical activity (minutes of moderate physical activity per week), behavioral outcomes (foot care, blood glucose monitoring, and medication taking), and glycemic targets and cardiovascular outcomes (HbA_1c_ levels, lipid levels, BP, BMI, and waist circumference) in adults with T2DM. Participants in this RCT were mainly middle-aged adults (mean age of 58.85, SD 10.14 years), and the majority (59.2%) were female. Although the sex distribution mirrors that found in clinical trials (57% female and 42% male [74]), this study had a far greater proportion of male participants (40.8%) than that usually found in web-based behavioral trials [75]. There were no statistically significant baseline demographic or behavioral differences (exception of diabetes support) in the participants between the diabetes self-management training WebControl group and the LIVE intervention group; therefore, the groups were well matched at baseline. Participants in both arms of the study were initially well distributed, with 102 participants in the LIVE group and 109 in the WebControl group. Although the number in each group decreased over time due to loss to follow-up, we used an intent-to-treat analysis and thus used all available data at various time points. It was very difficult to keep participants engaged for 12 months in a behavioral intervention. Learning from our first study [54] of not changing content after the site opened, we changed static educational content monthly within both the LIVE and WebControl sites (new grocery, restaurant, exercise, and bookstore content) and added new games weekly to only the LIVE site. We additionally provided leaderboards on both sites (the most frequent participants and the highest Fitbit users) using the principles of gamification as mechanisms to keep both groups engaged. We sent birthday cards and newsletters and additionally kept users engaged through forums, which enhanced on-going participation through reciprocity and cocreation and gave users a sense of ownership [76]. Participants tended to come to the first 12 to 26 weeks of classes, where the diabetes education content covered the American Diabetes Association and American Association of Diabetes Educators content in the first 12 weeks and followed by an advanced series developed by our diabetes educators in the following 13 weeks [61]. It was during this time that the participants slowly dropped out, and this may be due to the content being repeated in the next 26 weeks. Additionally, it should be noted that our activities on the site were retention driven and not participation driven. Our lesson learned with regard to diabetes education in both sites was that 12 months was most likely not necessary to begin to improve metabolic outcomes. Participants may have different preferences for synchronous versus asynchronous classes, and this is why we need to carefully determine the needs and learning preferences of persons with diabetes. Further analysis of the quality of participation including time spent, the amount of interaction with objects and others, and locations visited on both sites will be addressed in a future paper.

Overall, both groups benefitted over time. Both had access to a computer-based platform; however, the LIVE group was interactive and synchronous and the WebControl group was web based and asynchronous. We frequently changed and updated the content on both the LIVE site and the WebControl site to keep our participants engaged and interested. A good website is one that keeps the users engaged on the website, including keeping the content interactive and physically engaging using both the mouse and keyboard. This was certainly true for the LIVE site and the WebControl site, which provided many clickable items to view further information. We ensured that the design was effective, efficient, and satisfying to the users [53]. We additionally used gamification such as leaderboards to incentivize our users to interact with either site on a regular basis.

Of note, with the exception of weight and waist circumference, there were optimal glycemic targets at study onset. However, physical activity levels were light to moderate and there was low daily fruit and vegetable and fiber intake at study onset.

We compared the effects of the LIVE and WebControl groups on diet and found that our participants at baseline in both arms of the study had a daily percent energy from fat in the 30% range with the LIVE group reporting 35.36% and the WebControl group reporting 34.13%, although it was at the higher end of recommended amounts. Throughout the 12 months of the study, participants in both groups maintained a stable daily percent of energy from fat. The American Heart Association recommends a range of 20% to 35% of calories from fat daily [77]. These recommendations are based on reducing the risk of chronic diseases, ensuring essential daily nutrients, and having adequate energy intake [77].

The Institute of Medicine recommends that total daily dietary fiber intake should be 19-30 grams, depending on age and sex [78]. This recommendation is based on ensuring adequate daily nutrients and assisting with a fat-modified diet that helps to lower cholesterol [79]. However, dietary fiber intake in the United States is well below that of recommendation, with an average of 15 grams per day in those older than 20 years and an average of 17 grams in those aged 50-59 years [80]. In our study, fiber intake was below the recommended levels at baseline with mean values of 16.20 grams in the LIVE group and 17.16 grams in the WebControl group. The daily fiber intake increased in both groups from baseline to 6 months and then declined. However, there were no statistically significant changes within and between the groups with regard to fiber intake.

Consuming fruits and vegetables daily is an important part of reducing chronic diseases such as cardiovascular disease or T2DM. Most adults in the United States do not meet the recommended guidelines for servings of fruits and vegetables per day and the Centers for Disease Control and Prevention report a median of 2.65 servings of fruits and vegetables per day [81]. The American Heart Association recommends consuming 4 servings of fruits and 5 servings of vegetables per day [82]. In this study, the daily servings of fruits and vegetables were over the national average but remained far below the recommended daily amounts.

The US Department of Health and Human Services recommends at least 150-300 minutes or 2.5-5 hours of moderate-intensity physical activity per week [83]. A recent study showed that 4400 steps per day was significantly related to lower mortality rates in women and the benefits leveled off at 7500 steps per day [84]. A recent systematic review showed an inverse relationship between steps per day and cardiovascular events up to 10,000 steps per day [85]. In our study, participants self-reported exercise as days per week and tracked their steps via Fitbit in both groups. In the LIVE group, there was a small increase in number of steps per week and an increase in days of exercise per week from a median of 2.63 days at baseline to 3.40 days at 12 months. The results were similar in the WebControl group with an increase in days of exercise per week from a median of 2.67 days to 3.38 days. In the LIVE group, the median daily steps increased from 6153 steps at baseline to 6347 steps at 12 months. In the WebControl group, the median daily steps increased from 5570 steps at baseline to 6532 steps at 12 months. In this study, we encouraged participants to exercise by using Fitbit activity trackers and gamification. On a monthly basis, we posted the top 3 participants who walked the most steps on a leaderboard within the VE and the website. Although we did not see a significant difference between data at baseline and 12 months in either group, we did note a trend toward improvement in each group. Evidence shows how the use of Fitbits allows individuals to watch their daily number of steps and gamification assists individuals in becoming more motivated to engage in physical activity [86].

The majority of our participants had optimal HbA_1c_ levels at study onset with a mean HbA_1c_ level of 7.62% (SD 1.76%; LIVE group: mean 7.50%, SD 1.58%; WebControl group: mean 7.73%, SD 1.92%). Participants in both groups showed decreasing trends in HbA_1c_ levels over the 12 months in the study, although not significantly. The results are similar for BP with a mean value at baseline of 137/81 and no significant changes per group over the 12 months. All of the values for lipid levels at baseline were close to the recommended targets, so very little change was expected. We did show that more participants in the LIVE group lost weight than those in the WebControl group (*P*=.04). With regard to behavioral outcomes of foot care, blood glucose monitoring, and medication taking, participants in the LIVE group had a consistent improvement in all 3 outcomes over the 12 months compared to those in the WebControl group, which showed fluctuations over the 12 months.

Since most participants met glycemic targets at the beginning of the study, we examined changes in glycemic targets by randomized group among a subsample of participants whose HbA_1c_ level was greater than or equal to 8.6% at baseline. In both arms of the study, HbA_1c_ levels dropped from baseline to 12 months. There were no statistically significant changes per group over time; however, we did see trends in the improvement of glycemic targets in those who began the study with higher HbA_1c_ levels.

It must be taken into consideration that both groups had access to a content-tailored computer program, although the LIVE group was interactive and synchronous at times (classes given in-person by an avatar), whereas the WebControl group was asynchronous (classes were recorded). Since both groups had equal access to educational content, it is not surprising that both groups showed a decrease in most variables, thus meeting glycemic targets. The same was true for foot care and blood glucose monitoring.

We saw the most impact on those who had higher HbA_1c_ levels at baseline (>8.6%), which was an exploratory aim. There were clinical improvements in only the LIVE group from baseline to 12 months in systolic blood pressure, weight, and BMI. There were clinical improvements in only the WebControl group from baseline to 12 months in HDL. On average, HbA_1c_ levels improved in the LIVE group by 0.73% (SD 0.65%; range –2.0% to 1.7%) and in the WebControl group by 1.65% (SD 1.59%; range 0.4%-5.2%). The LIVE group dropped their HbA_1c_ levels on average by 1 percentage point, whereas the WebControl group dropped their HbA_1c_ levels on average by over 2 percentage points. A drop of 1 percentage point in HbA_1c_ level was found to significantly reduce diabetes-related microvascular complications [2]. This speaks to the use of offering web-based interventions to those who are facing challenges with glycemic targets.

In this 12-month study, we noticed some improved outcomes at 6 months, such as days of exercise per week and exercise intensity, percent of fat intake, fiber intake, fruit and vegetable intake, LDL, and days of blood glucose monitoring. We think that these results may be due to the fact that the first round of educational classes ended at approximately 26 weeks, and after that, the same classes were repeated for another 26 weeks. This study showed that people do not have to attend solely in-person classes to impact changes on health behaviors, as virtual connectivity offered health benefits. Certainly, many different types of telehealth interventions have had a positive impact on T2DM self-management health behaviors [14,87,88]. In future studies, we will examine the translation of this virtual platform to various health care settings or communities and other chronic conditions. Moving beyond an RCT will teach us important things about intervention efficacy in different populations outside of a self-selected research sample. Preferences for synchronous and asynchronous DSME/S may vary by person and over time when living with a chronic condition. Therefore, future research should explore outcomes among those who exhibit a need for intervention based on glycemic variability or stability and the effects when participants can choose the type of platform they engage in.

### Limitations

The LIVE study recruited a self-selected group of volunteers who had met their glycemic targets based on average HbA_1c_ levels (mean 7.64%, SD 1.79%) and had weight problems as measured by BMI. Participants who enrolled had a perceived need to volunteer for the study, perhaps to improve their overall health status or to learn about diabetes self-management in a virtual or web-based environment. Because the study premise was that VEs traverse geography and time and that the majority of persons with T2DM have weight management problems and typically have high HbA_1c_ levels, we did not enroll participants with suboptimal HbA_1c_ levels. The WebControl group received a web-based intervention that was more than they would have received on typical diabetes-focused websites or in the context of routine care, and thus, they benefitted, based on the majority of study parameters, in the same way that the LIVE participants did.

A main limitation is the decrease in data completion over the course of the 12 months of data collection. Engaging participants in behavioral longitudinal studies is difficult, particularly in-person follow-up for laboratory or clinical assessments. However, even the smaller number of participants with full longitudinal data provides us with outcomes that are clinically relevant and illustrates what we may anticipate in larger translational implementation of the LIVE intervention. The study findings also have limitations in that the majority of participants were female, non-Hispanic, and White individuals. We attempted to recruit a sample diverse in race, ethnicity, and sex by having a suburban/rural site and a major metropolitan city site. We cast a broad net for recruitment with multiple strategies; however, we found that particularly in the urban city site that individuals preferred in-person programs rather than virtual or web-based programs. It would be interesting to see how this might be different given changes in health care delivery, particularly telemedicine during and after the COVID-19 pandemic.

### Conclusion

T2DM is mainly a self-management condition. Since we know that frequent interaction between providers and persons with diabetes improves outcomes, our aim was to develop a tool where the providers and persons with diabetes could easily and frequently interact and the content within the environment could be easily changed by the providers depending on the needs of the persons with diabetes. This study confirmed that there were minor positive changes on glycemic targets in both groups over the 12-month study period; however, the majority of the participants began with optimal HbA_1c_ levels (mean 7.64%, SD 1.79%). We did find clinically relevant metabolic changes in those who began with an HbA_1c_ level >8.6% in both groups. It does appear that the LIVE group’s metabolic outcomes were a little better than those of the WebControl group. Taken as a whole, given that this study provided a variety of resources to our participants in both study groups, we suggest that a toolkit with a variety of services such as web-based, synchronously held classes and support groups; recorded classes; research updates; games; and interactive content and feedback could allow people to get what they need with regard to their individualized level of knowledge and learning styles.

## Appendix

Multimedia Appendix 1 CONSORT-eHEALTH checklist (V 1.6.1).

## Abbreviations

BP: blood pressure
CONSORT: Consolidated Standards of Reporting Trials
DSME/S: Diabetes self-management education and support
LDL: low-density lipoprotein
LIVE: Learning in a Virtual Environment
HbA_1c_: glycated hemoglobin
HDL: high-density lipoprotein
REDCap: Research Electronic Data Capture
RCT: randomized controlled trial
SLIDES: Second Life Impacts Diabetes Education & Support
T2DM: type 2 diabetes mellitus
VE: virtual environment
